# Special Issue: Development of Asymmetric Synthesis

**DOI:** 10.3390/molecules25061266

**Published:** 2020-03-11

**Authors:** Rafael Chinchilla

**Affiliations:** Departamento de Química Orgánica, Facultad de Ciencias, and Instituto de Síntesis Orgánica (ISO), Universidad de Alicante, P.O. Box 99, 03080 Alicante, Spain; chinchilla@ua.es

Biological systems usually respond differently to enantiomers of a chiral molecule due to the inherent chirality of the active receptor sites of enzymes in nature. Therefore, it highly desirable to obtain molecules in enantiomerically pure form to achieve, for instance, interesting pharmacological responses. For the synthesis of enantiomerically pure compounds, the chemist either can synthesize a racemic mixture and then resolve it into their enantiomers or, much more efficiently, develop a synthesis able to produce the desired compounds asymmetrically. Thus, asymmetric synthesis is an area of research which experiences continuous developments, from the direct modification of chiral molecules to the use of catalyzed reactions using chiral metal complexes or chiral metal-free organocatalysts. This Special Issue collects research articles showing significant examples of recent developments in the use of the existing different methodologies for the asymmetric synthesis of compounds of interest.

The use of an easily available initial enantiomerically pure compound as a template able to control the stereoselectivity of the reaction when preparing interesting enantiopure products is illustrated by Moriyama et al. [1]. Thus, (*R*)-glycidol is used as starting material for the preparation of the four chiral diastereomers of pestalotin, which naturally is a gibberellin synergist, using a straightforward and divergent manner based on the use of diastereoselective aldol and hetero-Diels–Alder reactions, as well as Mitsunobu inversions. Han et al. show a diastereoselective process specifically developed for the large-scale preparation of (*S*)-2-amino-4,4,4-trifluorobutanoic acid, a bioisostere of leucine moiety in drug design [2]. In this case, a chiral auxiliary forms a nickel(II) complex with a glycine Schiff base and is diastereoselectively alkylated with 2,2,2-trifluoroethyl iodide; the resulting complex is disassembled to give the recyclable chiral auxiliary and the fluorinated amino acid in a multigram scale.

The use of catalysts is nowadays the most frequently used procedure when asymmetric reactions are intended, as a small amount of a chiral species produces large quantities of an optically active compound from a precursor. Metal complexes bearing chiral ligands have been the most employed catalytic species in asymmetric transformations, and this Special Issue includes interesting examples. Thus, the article by Claros et al. shows the use of a family of enantiopure ruthenium complexes bearing phosphonite of phosphinite ligands in the catalytic asymmetric transfer hydrogenation of aryl ketones with isopropyl alcohol as a hydrogen source, obtaining in some cases high conversions and enantioselectivities of the corresponding benzyl alcohols [3]. Another example of the use of chiral metal complexes as catalysts in asymmetric synthesis is shown by Liu et al. [4]. In this case, a chiral nickel-aminophenol sulfonamide complex is employed as a catalyst for the asymmetric Henry reaction between 2-acylpyridine *N*-oxide-including ketones with nitromethane; the reaction is a challenge as *N*-oxides generally act as competitive catalyst inhibitors or displace the ligands.

In the last years, the use of organocatalysis in asymmetric synthesis has experienced growing interest due to some advantages compared to the use of metal complexes as catalysts, such as their usual higher stability, possible recyclability or lower ecological footprint due to the metal-free character of these purely organic catalysts. Thus, the use of these organocatalysts is also represented in this Special Issue, as is the case of the article by Manaprasertsak et al. which presents an efficient and economic preparation of different *C*_2_-symmetrical 3,3′-diaryl substituted tetranaphthobisazepinium bromides; such compounds have shown wide application as highly efficient chiral phase-transfer catalysts [5]. The obtained catalysts are employed in the asymmetric substitution of a glycine-derived Schiff base under phase-transfer conditions leading to enantioenriched amino acid derivatives. Another example of the use of asymmetric organocatalysis is shown in the article by Torregrosa-Chinillach et al. where a chiral primary amine-salicylamide is used as an organocatalyst for the enantioselective conjugate addition of α,α-disubstituted aldehydes to maleimides and nitroalkenes, using deep eutectic solvents as reaction media, with catalyst and solvent being recovered and reused [6]. These neoteric solvents are growing in popularity due to their environmental advantages, such as low toxicity, low volatility, and low price.

This Special Issue shows that the search for new methodologies suitable for achieving asymmetric synthesis remains quite active, as still more efficient stereoselective methodologies need to be developed. Asymmetric synthesis will continue to be a fast-moving research area in the coming years.
